# Using Venn Diagrams to Evaluate Digital Contact Tracing: Panel Survey Analysis

**DOI:** 10.2196/30004

**Published:** 2021-12-06

**Authors:** Paola Daniore, Vasileios Nittas, André Moser, Marc Höglinger, Viktor von Wyl

**Affiliations:** 1 Institute for Implementation Science in Healthcare University of Zurich Zurich Switzerland; 2 Epidemiology, Biostatistics and Prevention Institute University of Zurich Zurich Switzerland; 3 Clinical Trials Unit Bern University of Bern Bern Switzerland; 4 Winterthur Institute of Health Economics Zurich University of Applied Sciences Winterthur Switzerland

**Keywords:** digital contact tracing, exposure notification, COVID-19, SARS-CoV-2, contact tracing, digital health, tracing apps, mHealth, mobile apps, key performance indicators, Venn diagram approach

## Abstract

**Background:**

Mitigation of the spread of infection relies on targeted approaches aimed at preventing nonhousehold interactions. Contact tracing in the form of digital proximity tracing apps has been widely adopted in multiple countries due to its perceived added benefits of tracing speed and breadth in comparison to traditional manual contact tracing. Assessments of user responses to exposure notifications through a guided approach can provide insights into the effect of digital proximity tracing app use on managing the spread of SARS-CoV-2.

**Objective:**

The aim of this study was to demonstrate the use of Venn diagrams to investigate the contributions of digital proximity tracing app exposure notifications and subsequent mitigative actions in curbing the spread of SARS-CoV-2 in Switzerland.

**Methods:**

We assessed data from 4 survey waves (December 2020 to March 2021) from a nationwide panel study (COVID-19 Social Monitor) of Swiss residents who were (1) nonusers of the SwissCovid app, (2) users of the SwissCovid app, or (3) users of the SwissCovid app who received exposure notifications. A Venn diagram approach was applied to describe the overlap or nonoverlap of these subpopulations and to assess digital proximity tracing app use and its associated key performance indicators, including actions taken to prevent SARS-CoV-2 transmission.

**Results:**

We included 12,525 assessments from 2403 participants, of whom 50.9% (1222/2403) reported not using the SwissCovid digital proximity tracing app, 49.1% (1181/2403) reported using the SwissCovid digital proximity tracing app and 2.5% (29/1181) of the digital proximity tracing app users reported having received an exposure notification. Most digital proximity tracing app users (75.9%, 22/29) revealed taking at least one recommended action after receiving an exposure notification, such as seeking SARS-CoV-2 testing (17/29, 58.6%) or calling a federal information hotline (7/29, 24.1%). An assessment of key indicators of mitigative actions through a Venn diagram approach reveals that 30% of digital proximity tracing app users (95% CI 11.9%-54.3%) also tested positive for SARS-CoV-2 after having received exposure notifications, which is more than 3 times that of digital proximity tracing app users who did not receive exposure notifications (8%, 95% CI 5%-11.9%).

**Conclusions:**

Responses in the form of mitigative actions taken by 3 out of 4 individuals who received exposure notifications reveal a possible contribution of digital proximity tracing apps in mitigating the spread of SARS-CoV-2. The application of a Venn diagram approach demonstrates its value as a foundation for researchers and health authorities to assess population-level digital proximity tracing app effectiveness by providing an intuitive approach for calculating key performance indicators.

## Introduction

In recent efforts to limit the number of COVID-19 infections, a respiratory disease caused by SARS-CoV-2, digital proximity tracing apps have been deployed in multiple countries [1,2]. In Switzerland, the SwissCovid digital proximity tracing app complements conventional manual contact tracing procedures that are carried out by cantonal authorities to track the spread of SARS-CoV-2, regardless of whether an individual has the voluntary digital proximity tracing app installed or not [3]. Manual contact tracing, in the form of interviews of identified cases, is labor-intensive and prone to errors due to its reliance on individuals’ abilities to recall close-range proximity contacts [4]. Digital proximity tracing apps aim to overcome the limitations of manual contact tracing [5,6]. Most digital proximity tracing apps use Bluetooth low-energy beacons to track proximity contacts within a radius of 2 meters and notify individuals of a recent exposure to a SARS-CoV-2 clinically confirmed digital proximity tracing app user [7]. Digital proximity tracing apps promise to deliver notifications at a faster rate, with broader reach, and with greater scalability than manual contact tracing [5,8]. Their increased implementation in countries is widely associated with improved contact tracing and transmission mitigation in modeling studies [5,9,10].

There has been a surge of interest in evaluating the effectiveness of digital proximity tracing apps in mitigating the spread of SARS-CoV-2. Particular interest is placed on the added benefit of exposure notifications from digital proximity tracing apps relative to manual contact tracing in containing nonhousehold spread [11,12]. For example, recent studies in England [13] and Switzerland [14] revealed an average of 4 exposure notifications per index case were triggered by infected app users, which highlights the considerable breadth of digital proximity tracing apps over traditional manual contact tracing. However, attributing the contribution of digital proximity tracing apps on the mitigative actions taken by exposure notification recipients is a challenge due to inconsistent data availability [15]. This proves to be particularly challenging in countries such as Switzerland, where notified users of the digital proximity tracing app are not legally mandated to take action as a result of the exposure notification [16]. Furthermore, there is no unified methodology to evaluate the effectiveness of digital proximity tracing apps in mitigating the spread of infection. The lack of harmonization of terminology, indicator definitions, and monitoring goals has emerged in recent months as one of the key challenges in informing health policy about digital proximity tracing app effectiveness [8].

An approach based on Venn diagrams can be used to assess the effectiveness of digital proximity tracing apps in mitigating the spread of the SARS-CoV-2 infection. The use of Venn diagrams enables a structured approach to count outcomes across several subpopulations and for each period cross-sectionally. Very importantly, a Venn diagram approach also provides an intuitive framework for assessing generalizability and missing population segments of study data. Hence, when applied to population-level data, the approach enables the identification and construction of appropriate indicators in a reproducible manner, given the available data, to evaluate the impact of digital proximity tracing apps on users taking mitigative actions. The aim of this study was to demonstrate the use of Venn diagrams in assessing key indicators for exposure notification performance and effectiveness in mitigating the spread of SARS-CoV-2. We hope to guide health researchers and authorities in collecting relevant population-level data.

## Methods

### Overview

We applied a Venn diagram approach on data from COVID-19 Social Monitor, a nationwide panel study [17,18] of Swiss residents that allowed for the classification of (1) nonusers of the SwissCovid app, (2) users of the SwissCovid app and (3) users of the SwissCovid app who received exposure notifications, to provide a first description of the possible influence of exposure notifications on individuals taking mitigative actions against SARS-CoV-2 spread.

### SwissCovid Digital Proximity Tracing App

The Swiss digital proximity tracing app (SwissCovid app) was launched on June 25, 2020. The adoption of the app in the Swiss health care system and pandemic mitigation response has been described extensively in previous studies [14,19]. The SwissCovid app employs a decentralized privacy-preserving proximity tracing protocol [2], has been downloaded almost 3 million times [20] for a population of 8.4 million in Switzerland, and has an estimated 1.7 million active users. App users who receive exposure notifications are eligible for a free SARS-CoV-2 test and are instructed to call a federal information hotline. The hotline elicits further information about the possible risk exposure and determines if entering into quarantining is necessary, which happens in approximately 20% of cases [14]. The hotline can only recommend quarantine, which makes entering into quarantine voluntary, and individuals who choose to quarantine do not receive salary compensation. By contrast, mandatory quarantine can be ordered by cantonal health authorities or a physician through manual contact tracing, and individuals who are quarantined are entitled to salary compensation. As of March 2021, 60,000 app users who have tested positive have triggered exposure notifications, and 70,000 telephone calls to the information hotline have been logged [20].

### Venn Diagram Approach

This approach makes use of Venn diagrams to visualize the co-occurrence of SARS-CoV-2 outcomes of interest based on digital proximity tracing app use [21]. To construct the Venn diagrams, as recently formalized [21], requirements are established to define the appropriate data sets and time points, as well as identify subpopulations, to calculate digital proximity tracing app effectiveness. Based on our experience and extensive reporting of key indicators to mitigate the spread of SARS-CoV-2 [2,19,20], we proposed 4 attributes for Venn diagram development to facilitate the identification of subpopulations of interest: (1) having been tested for SARS-CoV-2, (2) having a positive SARS-CoV-2 test result, (3) having received an exposure notification, and (4) having entered into quarantine (Table 1).

**Table 1:** Descriptive attributes of Venn diagram subpopulations.

Group	Tested for SARS-CoV-2	Positive test for SARS-CoV-2	Exposure notification (exposure notification)	Quarantine	Remarks
A	True	True	False	False	N/A^a^
B	True	True	True	False	N/A
C	True	True	True	True	Tested positive; received exposure notification; quarantined
D	True	True	False	True	Tested positive; did not receive exposure notification; quarantined
E1	True	False	True	False	Tested negative; received exposure notification; not quarantined
E2	False	False	True	False	Not tested; received exposure notification; not quarantined
F1	True	False	True	True	Tested negative; received exposure notification; quarantined
F2	False	False	True	True	Not tested; received exposure notification; quarantined
G1	True	False	False	True	Tested negative; did not receive exposure notification; quarantined
G2	False	False	False	True	Not tested; did not receive exposure notification; quarantined
T	True	False	False	False	Tested negative; did not receive exposure notification; not quarantined
N	False	False	False	False	Not tested; did not receive exposure notification, not quarantined

^a^N/A: not applicable to the applied scope; all individuals who tested positive were immediately placed in isolation.

The allocation of individuals to each subsection, and their resulting overlap, enabled a more thorough definition of subpopulations. Once the subpopulations were identified, they were labeled according to the Venn diagram allocation. These labels facilitated the calculation of key digital proximity tracing effectiveness indicators by identifying relevant numerators and denominators (eg, the number of app users who received exposure notifications and entered into quarantine versus the number of app users who received exposure notifications but did not enter into quarantine). Furthermore, the data visualizations through Venn diagrams enabled an overview of available data, time horizons, as well as missing and available populations for analyses. A Venn diagram approach thereby provides a general methodology to facilitate the formulation of research hypotheses, aid the selection of suitable databases, and help to define key performance indicators by referencing to specific, labeled diagram segments. For our study, we defined a priori guiding criteria and definitions (Multimedia Appendix 1) to interpret Venn diagrams in the context of the SARS-CoV-2 pandemic in Switzerland. We also applied targeted questions (Multimedia Appendix 1) on digital proximity tracing app use in Switzerland from our panel survey to the Venn diagram population set.

### Data Collection

Data from COVID-19 Social Monitor [17,18] comprise a representative stratified random survey panel of 3381 participants from Switzerland. Participants were randomly sampled from an existing web-based market research panel from a Swiss survey company. The study was launched in March 2020, with 14 survey waves (as of March 2021) which have been offered every 4 to 6 weeks. The sample was replenished in December 2020 by recruiting new participants from the same market research panel to counteract panel attrition.

Our study used data from 4 survey waves: December 14 to December 23, 2020 (December survey); January 25 to February 4, 2021 (January survey); February 22 to March 3, 2021 (February survey); and March 29 to 08 April 8, 2021 (March survey). The December survey was used as the baseline for this study due to high SARS-CoV-2 incidence in Switzerland at that time, which averaged approximately 4000 newly detected COVID-19 cases daily and a test positivity rate of 16% [22]. This period encompasses the peak of the second COVID-19 wave in December 2020 and the subsequent decline of infections in the following months. The January, February, and March follow-up surveys were used to record information on new outcomes of interest. Only participants with at least one follow-up survey were included in our analyses. The first instance of an event of interest (eg, testing positive for SARS-CoV-2) was included in our analyses; after the event of interest, the participant was excluded from further analyses. Participants with a self-reported positive SARS-CoV-2 test prior to or at baseline were excluded in order to include only new infection cases in the assessment period. We also expected that individuals were likely to react differently to a first receipt of an exposure notification in comparison to future exposure notification receipts. Therefore, we also excluded participants who received exposure notifications prior to or at baseline. National SARS-CoV-2 incidence and test positivity were also extracted to provide context on the state of the pandemic at the time the surveys were conducted [22,23].

### Statistical Analysis

Descriptive statistics were calculated for survey respondent demographics and to assess mitigative actions taken by the participants within the study period. Analyses were performed on the full study sample, as well as across the 3 subgroups of digital proximity tracing app nonusers and digital proximity tracing app users who did or did not receive an exposure notification.

Performance measures were constructed on the basis of labeled Venn segments representing subpopulations with the attributes shown in Table 1. We assessed the proportion of individuals who received SARS-CoV-2 testing, tested positive for SARS-CoV-2 (including the percentage of individuals who tested positive for SARS-CoV-2 among those who were tested), or entered into quarantine at the request of health care professionals or health authorities. The assessment of these indicators was stratified according to the 3 subgroups defined by digital proximity tracing app use or nonuse and exposure notification receipt.

We assessed the mitigative actions taken by individuals who received an exposure notification. These actions included (1) calling an information hotline to obtain advice on appropriate actions, (2) getting tested after receiving an exposure notification, and (3) entering into quarantine, including entering into quarantine as recommended by health care professionals or ordered by health authorities. The assessed key indicators are derived from the subpopulations observed in the Venn diagram based on digital proximity tracing app use.

We report 95% confidence intervals based on an exact binomial test for proportions for the estimation of subpopulation sizes and key indicators. Analyses were performed in Stata (version 16.1; StataCorp LLC). Continuous variables were represented as median (interquartile range), and categorical variables were represented as count (percentage) with corresponding 95% confidence intervals.

### Ethics

For the COVID-19 Social Monitor project, the Ethics Committee of the Canton of Zurich confirmed that it does not fall under the Swiss Human Research Law (BASEC-Nr Req-2020-00323). This exemption was granted due to the fact that data were collected and treated anonymously throughout the project.

As per the decision of the Cantonal Ethics Commission of Zurich, explicit informed consent was not needed from participants for this particular study. However, participants gave their general permission to be part of research studies when accepting the invitation to the panel from which we sampled our respondents. Participation in the study was voluntary, and participants could withdraw from the study at any time.

## Results

### Study Population

**Figure 1:**
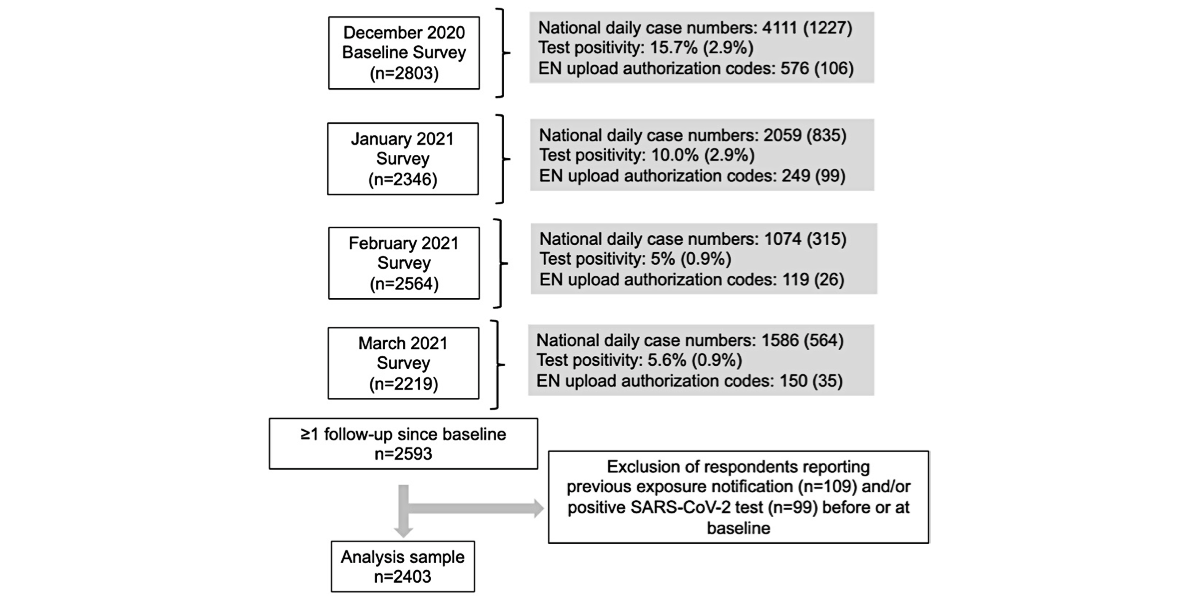
Flowchart of assessed panel survey data and associated SARS-CoV-2 incidence values. For the national daily case numbers, the values represent the daily average of SARS-CoV-2 cases in each month, with the values in parentheses representing their respective standard deviations. EN: exposure notification.

We included 12,525 assessments from 2403 participants in the final study cohort (Figure 1). The median respondent age was 49 years (IQR 35-59) and most (2305/2403, 95.9%) respondents were Swiss nationals (Table 2).

From the study cohort, 319 (319/2403, 13.3%) respondents reported having at least one of the following chronic illnesses: asthma, chronic obstructive pulmonary disease, diabetes, hypertension, cardiovascular disease, stroke, and cancer.

At the baseline survey (December 2020), 19.3% (236/1222) of app nonusers, 28.2% (325/1152) of app users who did not receive an exposure notification, and 34.5% (10/29) of app users who received an exposure notification reported an average monthly household income greater than 10,000 CHF (approximately US $10,886.43).

**Table 2:** Respondent demographics, self-reported health risks, and mitigative actions.

Characteristic		Full sample (n=2403)	Nonusers (n=1222)	App users (n=1152)	App users and received exposure notifications (n=29)
Age (in years), median (IQR)		49 (35-59)	49 (35-58)	49 (36-59)	40 (29-52)
**Gender, n (%)**					
	Female	1171 (48.7)	593 (48.5)	566 (49.1)	12 (41.4)
	Male	1232 (51.3)	629 (51.5)	586 (50.9)	17 (58.6)
**Personal status, n (%)**					
	No partner	668 (27.8)	353 (28.9)	310 (26.9)	5 (17.2)
	Living with partner	1518 (63.2)	755 (61.8)	739 (64.1)	24 (82.8)
	Not living with partner	217 (9.0)	114 (9.3)	103 (8.9)	0 (0.0)
**Family status, n (%)**					
	Has children	245 (10.2)	123 (10.1)	116 (10.1)	6 (20.7)
	Does not have children	2158 (89.8)	1099 (89.9)	1036 (89.9)	23 (79.3)
**Citizenship, n (%)**					
	Swiss	2173 (90.4)	1095 (89.6)	1051 (91.2)	27 (93.1)
	Swiss and other	132 (5.5)	72 (5.9)	59 (5.1)	1 (3.4)
	Non-Swiss	98 (4.1)	55 (4.5)	42 (3.6)	1 (3.4)
**Language region, n (%)**					
	German	1964 (81.7)	973 (79.6)	965 (83.8)	26 (89.7)
	French	274 (11.4)	160 (13.1)	113 (9.8)	1 (3.4)
	Ticino	165 (6.9)	89 (7.3)	74 (6.4)	2 (6.9)
**Education, n (%)**					
	Only mandatory schooling	64 (2.7)	42 (3.4)	22 (1.9)	0 (0.0)
	Completed professional education	1737 (72.3)	901 (73.7)	811 (70.4)	25 (86.2)
	University or university of applied sciences	602 (25.1)	279 (22.8)	319 (27.7)	4 (13.8)
**Employment status, n (%)**					
	Not working	721 (30.0)	370 (30.3)	344 (29.9)	7 (24.1)
	Currently working	1682 (70.0)	852 (69.7)	808 (70.1)	22 (75.9)
**Monthly household income (CHF^a^), n (%)**					
	≤6000	657 (27.3)	392 (32.1)	260 (22.6)	5 (17.2)
	6000-10,000	783 (32.6)	391 (32.0)	381 (33.1)	11 (37.9)
	>10,000	571 (23.8)	236 (19.3)	325 (28.2)	10 (34.5)
	No answer	392 (16.3)	203 (16.6)	186 (16.1)	3 (10.3)
**Health risks, n (%)^b^**					
	Smoker	507 (21.1)	287 (23.5)	211 (18.3)	9 (31.0)
	Self-reported chronic illness^c^	319 (13.3)	169 (13.8)	149 (12.9)	1 (3.4)
**Mitigative actions, n (%)^b^**					
	Always used protective masks when recommended	2335 (97.2)	1167 (95.5)	1140 (99.0)	28 (96.6)
	Always stayed at home except for essential tasks	1561 (65.0)	738 (60.4)	804 (69.8)	19 (65.5)
	Always kept recommended distance	2225 (92.6)	1103 (90.3)	1097 (95.2)	25 (86.2)
	Always refrained from visits	1583 (65.9)	743 (60.8)	824 (71.5)	16 (55.2)
Number of physical contacts, median (IQR)		3 (1-5)	3 (2-5)	3 (1-5)	3 (2-4)

^a^CHF: Swiss franc; At the time of publication, the exchange rate was approximately US $1=0.92 CHF.

^b^More than one or no answer is possible; therefore, percentages in this category do not add to 100%.

^c^At least one of the following chronic illnesses: asthma, chronic obstructive pulmonary disease, diabetes, hypertension, cardiovascular disease, stroke, or cancer.

### Baseline Adherence to Mitigation Strategies

During the high-incidence period in December 2020 (ie, baseline), most participants reported consistent adherence, as opposed to occasional or no adherence, to wearing protective masks (2335/2403, 97.2%) and maintaining appropriate distance (2225/2403, 92.6%) (Table 2). Despite the strongly recommended restrictions on mobility imposed in winter 2020 throughout Switzerland, 1561 (1561/2403, 65%) respondents reported leaving their households for nonessential tasks.

**Figure 2:**
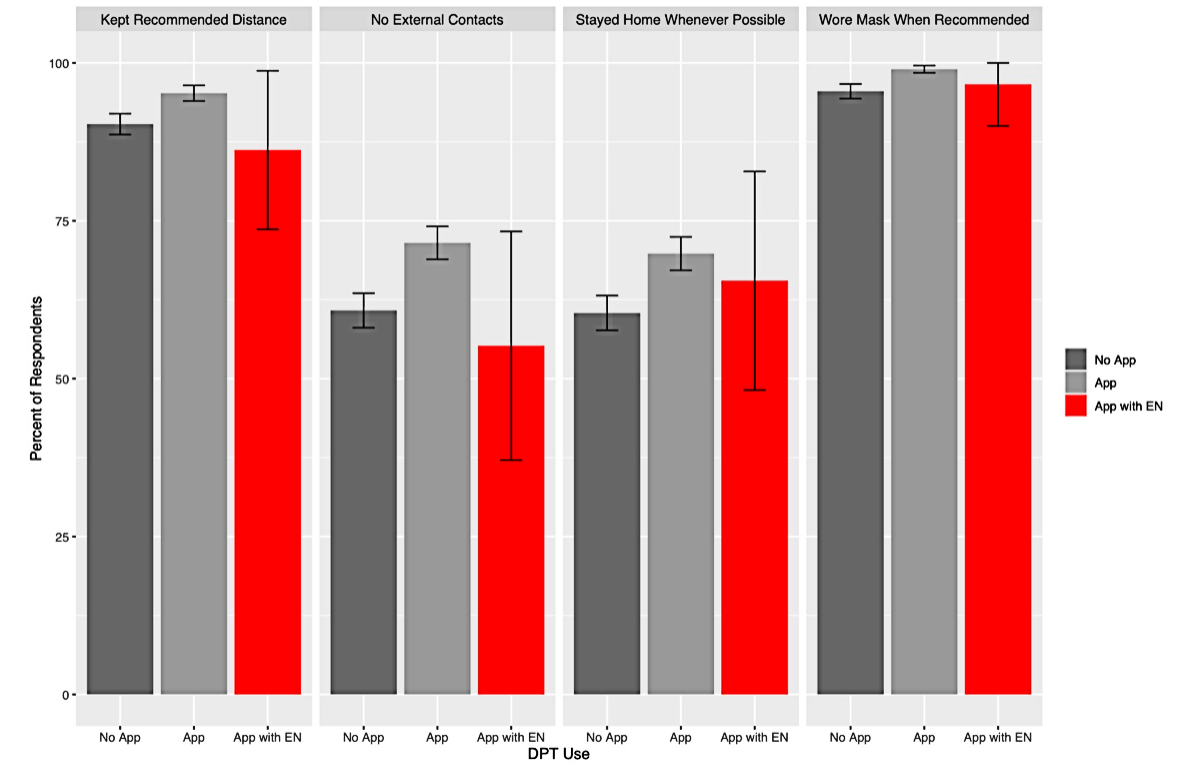
Baseline respondent mitigative actions based on reported SwissCovid app use with 95% confidence intervals. DPT: digital proximity tracing; EN: exposure notifications.

On average, 76.8% (938/1222) of app nonusers, 83.9% (966/1152) of app users who did not receive an exposure notification, and 75.9% (22/29) of app users who received an exposure notification reported adherence to at least one of the 4 mitigative measures (Figure 2).

### Population Sizes of Different Venn Diagram Segments

**Figure 3:**
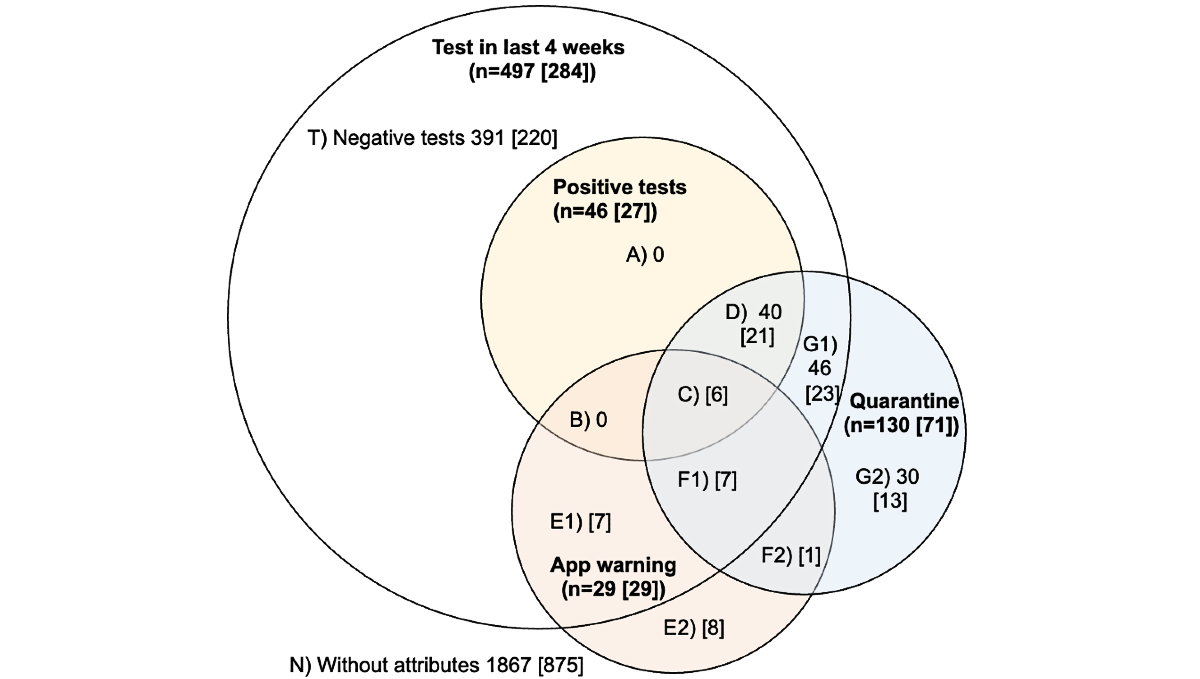
Venn diagram representation of mitigative actions taken by 4 survey subpopulations (in bold) after follow-up: (1) respondents who were tested for SARS-CoV-2 in the past 4 weeks (white circle), (2) respondents who tested positive for SARS-CoV-2 (yellow circle), (3) respondents who received exposure notifications (red circle), and (4) respondents who were placed in quarantine by Swiss cantonal health services or by a physician (blue circle). Sample sizes of specific segments are indicated in the diagram, with the values in [square brackets] reflecting the number of DPT app users in a given segment. Each (non)overlap represents a subpopulation of the survey respondents.

Based on outcomes reported across the 4 survey waves from December 2020 to March 2021, the respondent sample is visualized in a Venn diagram according to 4 categories (Figure 3). The corresponding subpopulation sizes for each Venn diagram segment are shown in Table 3. The sample is further divided into the 3 respondent subgroups based on digital proximity tracing app use and receipt of exposure notifications in order to calculate subgroup-specific indicators. Of note, segments A and B are empty by design, as all positive tested individuals are reported immediately to health authorities who, in turn placed these individuals in isolation (Table 3).

Overall (of the 2403 respondents), 46 (1.9%) respondents tested positive for SARS-CoV-2 in the follow-up period, 29 (1.2%) received exposure notifications, and 130 (5.4%) were placed into quarantine. In segment C, 6 (6/46, 13%) respondents who tested positive for SARS-CoV-2 reported having received an exposure notification. In segments E1 and F1 (14/29, 48.3%) were respondents who received exposure notifications and who tested negative for SARS-CoV-2, of whom 7 (7/29, 24.1%) respondents in segment F1 were placed into quarantine after having received exposure notifications. By contrast, in segments E2 and F2, 9 (9/29, 31%) respondents who received exposure notifications were not tested for SARS-CoV-2; the respondent in F2 was still placed into quarantine (Figure 3).

**Table 3:** Subpopulation cumulative mitigative actions and outcomes from the Venn diagram based on SwissCovid app use after respondent follow-up.

Group^a^	Nonusers, n	App users, n		All (n=2403), n	Percentage of entire sample (95% CI)
		No notifications	Received exposure notification		
C	0	0	6	6	0.2 (0.1-0.5)
D	19	21	0	40	1.7 (1.2-2.3)
E1	0	0	7	7	0.3 (0.1-0.6)
E2	0	0	8	8	0.3 (0.1-0.7)
F1	0	0	7	7	0.3 (0.1-0.6)
F2	0	0	1	1	0.04 (0.001-0.02)
G1	23	23	0	46	1.9 (1.4-2.5)
G2	17	13	0	30	1.2 (0.8-1.8)
N	992	875	0	1867	77.7 (76.0-79.3)
T	171	220	0	391	16.3 (14.8-17.8)

^a^The letters correspond to the subpopulations in the Venn diagram in Figure 3.

### Indicators Derived From Venn Diagrams

Denominators of subpopulations were selected to assess key indicators of SwissCovid app performance in reducing the spread of SARS-CoV-2 based on the mitigative actions taken and outcomes of interest by respondent groups (Table 4).

Overall, 49.2% (95% CI 47.1%-51.2%) of all participants reported using the SwissCovid app in at least one follow-up survey. App users contributed to 57.1% (95% CI 52.7%-61.5%) of SARS-CoV-2 tests taken, 58.7% (95% CI 43.2%-73%) of positive SARS-CoV-2 tests and 54.6% (95% CI 45.7%-63.4%) of respondents who entered into quarantine in our sample.

Test positivity among those who sought SARS-CoV-2 testing was 8.9% (95% CI 5.5%-13.6%) among app nonusers, 8% (95% CI 5%-11.9%) among app users who did not receive exposure notifications, and 30% (95% CI 11.9%-54.3%) among app users who received exposure notifications.

Entering into quarantine was reported by 4.8% (95% CI 3.7%-6.2%) of the app nonusers, 5% (95% CI 3.8%-6.4%) of the app users who did not receive exposure notifications, and 48.3% (95% CI 29.5-67.5%) of the app users who received exposure notifications. Similarly, entering into quarantine following testing for SARS-CoV-2 was reported by 19.7% (95% CI 14.6%-25.7%) of the app nonusers, by 16.7% (95% CI 12.4%-21.7%) of the app users who did not receive exposure notifications, and by 65% (95% CI 40.8%-84.6%) of the app users who received exposure notifications.

The percentage of respondents who received exposure notifications among those who tested positive (22.2%, 95% CI 8.6%-42.3%) for SARS-CoV-2 at a later point in time was higher than app users who tested negative (5.5%, 95% CI 3%-9%).

**Table 4:** Selection of appropriate numerators and denominators from a Venn diagram based on SwissCovid digital proximity tracing app use after respondent follow-up. Letters in square brackets—[]—reflect subpopulations (Venn segments) of app users and those in curly brackets—{}— reflect the subset of individuals who did not use the app.

Indicator			Numerator		Denominator		% (95% CI)
**Coverage of app users**							
	All	[C,D,E1,E2,F1,F2,G1,G2,N,T]		C,D,E1,E2,F1,F2,G1,G2,N,T		49.15 (47.13-51.17)	
	Tested	[C,D,E1,F1,G1,T]		C,D,E1,F1,G1,T		57.14 (52.66-61.54)	
	Tested positive	[C,D]		C,D		58.70 (43.23-73.00)	
	Quarantined	[C,D,F1,F2,G1,G2]		C,D,F1,F2,G1,G2		54.62 (45.65-63.36)	
**Individuals tested**							
	Nonuser	{D,G1,T}		{D,G1,G2,N,T}		17.43 (15.34-19.68)	
	App user	[D,G1,T]		[D,G1,G2,N,T]		22.92 (20.52-25.45)	
	App user and received exposure notifications	[C,E1,F1]		[C,E1,E2,F1,F2]		68.97 (49.17-84.72)	
**Test positivity**							
	Nonuser	{D}		{D,G1,T}		8.92 (5.46-13.58)	
	App user	[D]		[D,G1,T]		7.95 (4.99-11.90)	
	App user and received exposure notifications	[C]		[C,E1,F1]		30.00 (11.89-54.28)	
**Quarantined**							
	Nonuser	{D,G1,G2}		{D,G1,G2,N,T}		4.83 (3.70-6.18)	
	App user	[D,G1,G2]		[D,G1,G2,N,T]		4.95 (3.77-6.36)	
	App user and received exposure notifications	[C,F1,F2]		[C,E1,E2,F1,F2]		48.28 (29.45-67.47)	
**Quarantine (among those tested)**							
	Nonuser	{D,G1}		{D,G1,T}		19.72 (14.60-25.70)	
	App user	[D,G1]		[D,G1,T]		16.67 (12.38-21.72)	
	App user and received exposure notifications	[C,F1]		[C,E1,F1]		65.00 (40.78-84.61)	
App users who received an exposure notification who later tested positive			[C]		[C,D]		22.22 (8.62-42.26)
App users who received an exposure notification who later tested negative			[E1,F1]		[E1,F1, G1,T]		5.45 (3.01-8.97)

### Recommended Actions Taken Upon Receipt of Exposure Notifications

Recommended mitigative actions taken among respondents who received an exposure notification (segments C, E1, E2, F1, F2, n=29) are reported in Multimedia Appendix 1.

**Figure 4:**
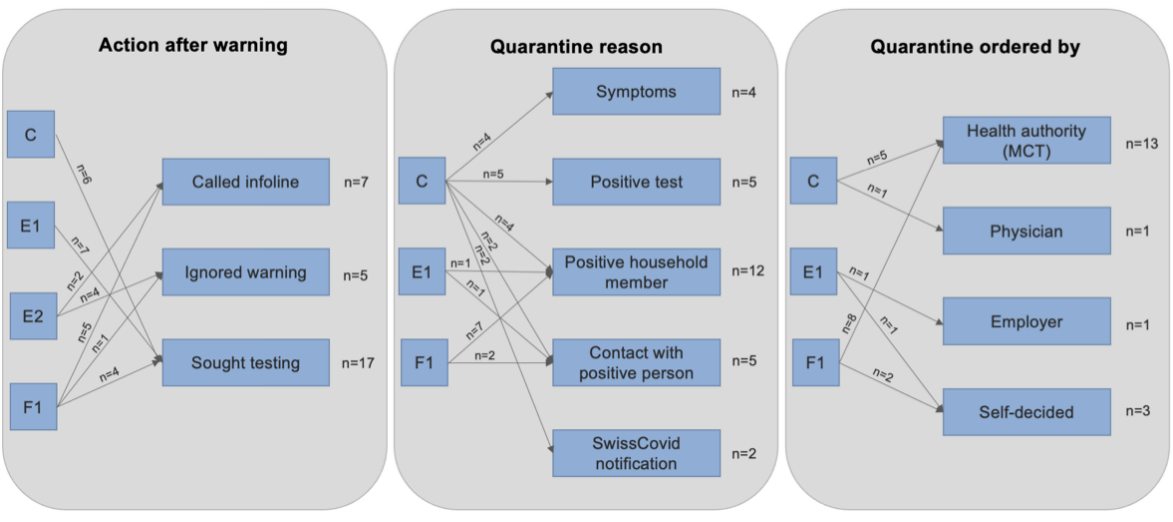
Tree diagram of subpopulation mitigative actions after follow-up in response to exposure notifications from the SwissCovid app. MCT: manual contact tracing.

Most respondents (17/29, 58.6%) who received exposure notifications sought SARS-CoV-2 testing and 7 (7/29, 24.1%) respondents called the federal information hotline. From these respondents, 22 (22/29, 75.9%) who received an exposure notification undertook at least one recommended mitigative action, while 5 (5/29, 17.2%) respondents explicitly stated to have ignored the exposure notification (Figure 4).

In group C, all 6 individuals reported to have sought testing after receiving an exposure notification: 4 (4/6, 66.7%) respondents reported having symptoms, and 2 (2/6, 33.3%) respondents reported entering into quarantine in response to the exposure notifications, even though one of these respondents reported not having symptoms.

Having contact with positive tested individuals or household members was almost always cited as a quarantine reason in groups C, F1, and F2 (with 1 exception). However, 5 (71.4%) out of 7 individuals in group E1 did not report possible contacts to positive tested individuals as a quarantine reason and yet still sought testing for SARS-CoV-2.

## Discussion

We were able to isolate subpopulations of interest and define performance indicators for digital proximity tracing app effectiveness. From our assessment, we found that a greater proportion of digital proximity tracing app users who received an exposure notification tested positive, in comparison to digital proximity tracing app users who did not receive an exposure notification and digital proximity tracing app nonusers. Our findings also suggest that the receipt of exposure notifications may contribute to SARS-CoV-2 transmission mitigation, as observed with most users from our cohort who voluntarily sought testing or called the federal information hotline, while half of these users received recommendations to self-isolate or quarantine as a result of manual contact tracing. Possible transmission mitigation was also observed in respondents who sought testing and who tested positive after receiving exposure notifications.

A previous cross-sectional analysis [17] of the same database revealed similar differences between app users and those who do not use digital proximity tracing apps to those in our study, namely with respect to citizenship status, household income, and adherence to mask-wearing. Furthermore, findings on user response to exposure notifications from our study complement those from an experimental study [24] in Spain that simulated exposure notification cascades. The findings of the study [24] suggested that 10% of individuals who received exposure notifications called the federal hotline. In our study, follow-up was sought by 24.1% (7/29) of digital proximity tracing app users who called the federal hotline after receiving exposure notifications..

Subpopulations visible in segments E1 (ie, individuals who tested negative, did not enter into quarantine and received an exposure notification) and E2 (ie, individuals who did not get tested, did not enter into quarantine and received an exposure notification) are currently not captured by any official statistics in Switzerland. Tracking responses to exposure notifications is challenging in Switzerland, since no data are systematically collected on individuals seeking testing after they have received exposure notifications, such as recording the reason for testing [25]. Responses to exposure notifications are also voluntary in Switzerland, making outgoing exposure notification data from the SwissCovid app inconclusive regarding the actual resulting mitigative measures taken by the users. In our study, our results suggest that approximately 1 in 2 individuals who receive exposure notifications may remain undetected and approximately 1 in 4 individuals do not respond to exposure notifications.

Positive test results for SARS-CoV-2 among app users who did not receive exposure notifications (8%, 95% CI 5%-11.9%) nonusers were similar (8.9%, 95% CI 5.5-13.6%) and of comparable magnitude to the officially reported test positivity values in Switzerland [22]. Notably, test positivity among app users who received exposure notifications was more than 3 times higher (30%, 95% CI 11.9%-54.3%). A recent report [26] also revealed similar findings of test positivity among app users in the Netherlands, which were recently found to be higher for users who received exposure notifications in comparison to those who did not. This raises the question of whether exposure notifications are reflective of an increased exposure risk [27]. Addressing this question is particularly relevant due to concerns that Bluetooth technology may not be able to capture exposure risks accurately [28,29]. By using data on digital proximity tracing app use with Venn diagrams, we presented a novel approach that evaluates the effect of exposure notifications on users’ mitigative actions as well as the risk of SARS-CoV-2 transmission through test positivity. However, such interpretations must account for national risk scoring schemes to allow for the identification of relevant exposure notifications. Switzerland, as an example, operates on conservative Bluetooth attenuation signal thresholds [3], whereas the United Kingdom has recently adopted lower thresholds in order to capture more exposures [30].

Our study also illustrates the usability and value of a Venn diagram approach to contextualize population- or survey-based evaluations of exposure notifications. We find that this method, and the extensive database used in this approach, provide a visual and analytical basis for assessing digital proximity tracing app effectiveness. Despite being based on over 12,500 follow-up surveys, our outcomes of interest such as SARS-CoV-2 infections (46/2403, 1.9%) and exposure notifications (29/2403,1.2%) were relatively rare. Nevertheless, the sample is likely well reflective of the population of app users as the sociodemographic characteristics associated with a higher propensity for electronic survey participation and SwissCovid app use likely overlap [17]. In contrary to the findings of another study [31], however, our database managed to cover all relevant segments of the Venn diagram, including groups of exposed contacts who received exposure notifications but did not respond to the warning. With even larger sample sizes, researchers can fulfill large enough subpopulation sizes to generate possible direct inference on digital proximity tracing effectiveness from the associated denominators. A current approach to evaluate the role of digital proximity tracing in the SARS-CoV-2 pandemic was presented in a recent study on factors associated with app use in Switzerland [17] and their associated effectiveness on the app notification cascade [14,19]. Another recent study [13] conducted in the United Kingdom also traced exposure notifications to a substantial number of individuals with nonhousehold risk exposures. By providing subpopulation-level granularity, a Venn diagram approach complements current evaluations of the role of digital proximity tracings in curbing SARS-CoV-2 transmission. This is particularly relevant in countries similar to Switzerland, where exposure notifications do not mandate mitigative actions from users [16].

## Limitations

Our study has some limitations. Due to the scarcity of relevant digital proximity tracing–related exposure notification outcomes, our study had limited statistical power. Owing to the mode of data collection (web-based panel surveys), the respondents in our sample may reflect subpopulations with above-average literacy and, possibly, higher adherence to recommended preventive actions against the SARS-CoV-2 infection. Therefore, our results might not be generalizable to the broader Swiss population. Also, while survey respondents are provided full anonymity, partial non- or overreporting of having received exposure notifications, of SARS-CoV-2 testing and positivity, as well as of nonadherence to measures, might have occurred. Furthermore, considering the small sample size of participants who received an exposure notification, findings of possible associated mitigative responses should be viewed as preliminary. Nevertheless, given the privacy-preserving nature of digital proximity tracing app design, survey-based exposure notification studies are among the few sources of data available to make assessments on their effectiveness in mitigating the spread of infection. As such, despite the limitations presented by surveys in including participants who received exposure notifications, our results are some of the first available to provide quantitative insights on the contribution of exposure notifications in digital proximity tracing app users taking mitigative actions. Lastly, the panel data did not provide enough granularity to recreate the full cascade sequence of risk exposure. Rather, the panel survey focused on gathering information on digital proximity tracing usage and associated mitigative actions yet not information on the premise surrounding any possible exposure notifications. Therefore, our data cannot univocally demonstrate causality of exposure notifications and SARS-CoV-2 transmission prevention.

## Conclusion

In our paper, we presented the Venn diagram as a tool to facilitate and streamline the evaluation of the role of digital proximity tracing apps in curbing the spread of the SARS-CoV-2 virus. By isolating subpopulations through a Venn diagram approach, a higher proportion of digital proximity tracing app users who tested positive after receiving an exposure notification was observed in comparison to digital proximity tracing app users who did not receive an exposure notification or digital proximity tracing app nonusers. Our findings also revealed that more than 3 out of 4 digital proximity tracing app users who received exposure notifications performed at least one recommended mitigative action, such as seeking SARS-CoV-2 testing or calling a federal information hotline, while half of these users received a recommendation to self-isolate or quarantine. An assessment, using a Venn diagram approach, of a larger population than the one presented in our study would allow the opportunity to assess the effectiveness of digital proximity tracing apps on users taking mitigative actions and their associated exposure risk with greater statistical power. This could, in turn, assist health authorities and researchers in identifying possible areas of improvement for digital proximity tracing apps alone, or in combination with manual contact tracing, by assessing effectiveness in curbing the spread of infection in a reproducible manner.

## Appendix

Multimedia Appendix 1 Subpopulation mitigative actions, Venn diagram analysis, and standardized questions on SwissCovid app use.

## Abbreviations

CHF: Swiss franc

## References

[1] Dar AB, Lone AH, Zahoor S, Khan AA, Naaz R (2020). Applicability of mobile contact tracing in fighting pandemic (COVID-19): issues, challenges and solutions. Comput Sci Rev.

[2] Troncoso C, Payer M, Hubaux JP, Salathé M, Larus J, Bugnion E, Lueks W, Stadler T, Pyrgelis A, Antonioli D, Barman L, Chatel S, Paterson K, Capkun S, Basin D, Beutel J, Jackson D, Roeschlin M, Leu P, Preneel B, Smart N, Abidin A, Gürses S, Veale M, Cremers C, Backes M, Tippenhauer NO, Binns R, Cattuto C, Barrat A, Fiore D, Barbosa M, Oliveira R, Pereira J Decentralized privacy-preserving proximity tracing. arXiv.

[3] Coronavirus: SwissCovid app and contact tracing. Swiss Federal Office of Public Health.

[4] Hellewell J, Abbott S, Gimma A, Bosse NI, Jarvis CI, Russell TW, Munday JD, Kucharski AJ, Edmunds WJ, Funk S, Eggo RM (2020). Feasibility of controlling COVID-19 outbreaks by isolation of cases and contacts. Lancet Glob Health.

[5] Ferretti L, Wymant C, Kendall M, Zhao L, Nurtay A, Abeler-Dörner L, Parker M, Bonsall D, Fraser C (2020). Quantifying SARS-CoV-2 transmission suggests epidemic control with digital contact tracing. Science.

[6] Kucharski AJ, Klepac P, Conlan AJK, Kissler SM, Tang ML, Fry H, Gog JR, Edmunds WJ, Emery JC, Medley G, Munday JD, Russell TW, Leclerc QJ, Diamond C, Procter SR, Gimma A, Sun FY, Gibbs HP, Rosello A, van Zandvoort K, Hué S, Meakin SR, Deol AK, Knight G, Jombart T, Foss AM, Bosse NI, Atkins KE, Quilty BJ, Lowe R, Prem K, Flasche S, Pearson CAB, Houben RMGJ, Nightingale ES, Endo A, Tully DC, Liu Y, Villabona-Arenas J, O'Reilly K, Funk S, Eggo RM, Jit M, Rees EM, Hellewell J, Clifford S, Jarvis CI, Abbott S, Auzenbergs M, Davies NG, Simons D (2020). Effectiveness of isolation, testing, contact tracing, and physical distancing on reducing transmission of SARS-CoV-2 in different settings: a mathematical modelling study. Lancet Infect Dis.

[7] Braithwaite I, Callender T, Bullock M, Aldridge RW (2020). Automated and partly automated contact tracing: a systematic review to inform the control of COVID-19. Lancet Digit Health.

[8] von Wyl V, Bonhoeffer S, Bugnion E, Puhan MA, Salathé M, Stadler T, Troncoso C, Vayena E, Low N (2020). A research agenda for digital proximity tracing apps. Swiss Med Wkly.

[9] Cencetti G, Santin G, Longa A, Pigani E, Barrat A, Cattuto C, Lehmann S, Salathé M, Lepri B (2021). Digital proximity tracing on empirical contact networks for pandemic control. Nat Commun.

[10] Moreno López JA, Arregui García B, Bentkowski P, Bioglio L, Pinotti F, Boëlle P, Barrat A, Colizza V, Poletto C (2021). Anatomy of digital contact tracing: role of age, transmission setting, adoption, and case detection. Sci Adv.

[11] Zastrow M (2020). Coronavirus contact-tracing apps: can they slow the spread of COVID-19?. Nature.

[12] Servick K (2020). COVID-19 contact tracing apps are coming to a phone near you. How will we know whether they work?. Science.

[13] Wymant Chris, Ferretti Luca, Tsallis Daphne, Charalambides Marcos, Abeler-Dörner Lucie, Bonsall David, Hinch Robert, Kendall Michelle, Milsom Luke, Ayres Matthew, Holmes Chris, Briers Mark, Fraser Christophe (2021). The epidemiological impact of the NHS COVID-19 app. Nature.

[14] Menges D, Aschmann H, Moser A, Althaus C, von Wyl Viktor (2021). A Data-Driven Simulation of the Exposure Notification Cascade for Digital Contact Tracing of SARS-CoV-2 in Zurich, Switzerland. JAMA Netw Open.

[15] Kretzschmar ME, Rozhnova G, Bootsma MCJ, van Boven M, van de Wijgert JHHM, Bonten MJM (2020). Impact of delays on effectiveness of contact tracing strategies for COVID-19: a modelling study. Lancet Public Health.

[16] (2020). Digital proximity tracing. Swiss National COVID-19 Science Task Force.

[17] von Wyl V, Höglinger M, Sieber C, Kaufmann M, Moser A, Serra-Burriel M, Ballouz T, Menges D, Frei A, Puhan MA (2021). Drivers of acceptance of COVID-19 proximity tracing apps in Switzerland: panel survey analysis. JMIR Public Health Surveill.

[18] Moser A, Carlander M, Wieser S, Hämmig O, Puhan MA, Höglinger M (2020). The COVID-19 Social Monitor longitudinal online panel: real-time monitoring of social and public health consequences of the COVID-19 emergency in Switzerland. PLoS One.

[19] von Wyl V (2021). Challenges for nontechnical implementation of digital proximity tracing during the COVID-19 pandemic: media analysis of the SwissCovid app. JMIR Mhealth Uhealth.

[20] SwissCovid app monitoring. Federal Statistical Office.

[21] Lueks W, Benzler J, Bogdanov D, Kirchner G, Lucas R, Oliveira R, Preneel B, Salathé M, Troncoso C, von Wyl V (2021). Toward a common performance and effectiveness terminology for digital proximity tracing applications. Front Digit Health.

[22] Coronavirus: situation in Switzerland. Swiss Federal Office of Public Health.

[23] COVID-⁠19 Switzerland. Federal Statistical Office.

[24] Rodríguez P, Graña S, Alvarez-León EE, Battaglini M, Darias FJ, Hernán MA, López R, Llaneza P, Martín MC, Ramirez-Rubio O, Romaní A, Suárez-Rodríguez B, Sánchez-Monedero J, Arenas A, Lacasa L (2021). A population-based controlled experiment assessing the epidemiological impact of digital contact tracing. Nat Commun.

[25] Daniore P, Ballouz T, Menges D, von Wyl V (2021). ​The SwissCovid Digital Proximity Tracing App after one year: were expectations fulfilled?. Swiss Med Wkly.

[26] Factsheet CoronaMelder. the Netherlands Rijksoverheid.

[27] Smyth B (2021). Estimating exposure risk to guide behaviour during the SARS-COV2 pandemic. Front Digit Health.

[28] Sattler F, Ma J, Wagner P, Neumann D, Wenzel M, Schäfer R, Samek W, Müller KR, Wiegand T (2020). Risk estimation of SARS-CoV-2 transmission from bluetooth low energy measurements. NPJ Digit Med.

[29] Soldano GJ, Fraire JA, Finochietto JM, Quiroga R (2021). COVID-19 mitigation by digital contact tracing and contact prevention (app-based social exposure warnings). Sci Rep.

[30] NHS COVID-19 app statistics. National Health Service COVID-19 app support.

[31] Ballouz Tala, Menges Dominik, Aschmann Hélène E, Domenghino Anja, Fehr Jan S, Puhan Milo A, von Wyl Viktor (2021). Adherence and association of digital proximity tracing app notifications with earlier time to quarantine: results from the Zurich SARS-CoV-2 cohort study. Int J Public Health.

