# Supplementary material for: Using Venn Diagrams to Evaluate Digital Contact Tracing: Panel Survey Analysis
Source: JMIR Public Health Surveill. 2021 Dec 6;7(12):e30004. doi: 10.2196/30004 (PMC8658229; doi:10.2196/30004)
Supplement: Multimedia Appendix 1 [file publichealth_v7i12e30004_app1.pdf]

Multimedia Appendix 1

Supplementary Table S1. Subpopulation mitigative actions followed by an exposure notification from respondents with available follow-up information about SwissCovid app use. MCT: manual contact tracing; EN: exposure notification.

		Action after warning			Quarantine Reason					Quarantine ordered by			
ID	Group	Called infoline	Ignored warning	Sought testing	Symptoms	Positive test	Positive household member	Contact with pos. person	SwissCovid notification	Health authority (MCT)	Physician	Employer	Self-decided
1	C	-	-	1	-	1	-	1	1	1	-	-	-
2	C	-	-	1	1	1	1	1	-	1	-	-	-
3	C	-	-	1	1	1	1	-	-	1	-	-	-
4	C	-	-	1	-	1	1	-	-	-	1	-	-
5	C	-	-	1	1	-	-	-	-	1	-	-	-
6	C	-	-	1	1	1	1	-	1	1	-	-	-
7	E1	-	-	1	-	-	-	-	-	-	-	-	-
8	E1	-	-	1	-	-	-	-	-	-	-	-	-
9	E1	-	-	1	-	-	-	1	-	-	-	1	-
10	E1	-	-	1	-	-	1	-	-	-	-	-	1
11	E1	-	-	1	-	-	-	-	-	-	-	-	-
12	E1	-	-	1	-	-	-	-	-	-	-	-	-
13	E1	-	-	1	-	-	-	-	-	-	-	-	-
14	E2	-	1	-	-	-	-	-	-	-	-	-	-
15	E2	-	1	-	-	-	-	-	-	-	-	-	-
16	E2	-	-	-	-	-	-	-	-	-	-	-	-
17	E2	-	1	-	-	-	-	-	-	-	-	-	-
18	E2	1	-	-	-	-	-	-	-	-	-	-	-
19	E2	-	-	-	-	-	-	-	-	-	-	-	-
20	E2	-	1	-	-	-	-	-	-	-	-	-	-
21	E2	1	-	-	-	-	-	-	-	-	-	-	-
22	F1	1	-	-	-	-	1	1	-	1	-	-	-
23	F1	1	-	1	-	-	1	-	-	1	-	-	-
24	F1	-	-	1	-	-	1	-	-	1	-	-	1
25	F1	1	-	1	-	-	1	-	-	1	-	-	-
26	F1	-	1	-	-	-	1	-	-	1	-	-	1
27	F1	-	-	1	-	-	-	1	-	1	-	-	-
28	F1	1	-	-	-	-	1	-	-	1	-	-	-
29	F2	1	-	-	-	-	1	-	-	1	-	-	-
N	29	7	5	17	4	5	12	5	2	13	1	1	3
% EN recipients	100	24.1%	17%	59%	14%	17%	41%	17%	7%	45%	3%	3%	10%

Supplementary Table S2. Outline and purpose of the Venn diagram analysis.

**Analysis aspects**	**Definition**
Analysis Goal: What type of high-level task or metric should be described by the Venn diagram?	To place the sub-population of individuals with exposure notifications in the larger population context.
Population: What is the origin of the data (case series, cohort study, population-based study, administrative database)?	The online surveys were conducted within a market research panel. The study population was selected to be representative for Switzerland with respect to the distribution of age, gender, and language region.
Time horizon: What is the time perspective covered by the Venn diagram (cross-sectional, cumulative over a longer period)?	Data show cumulative events over four follow-up surveys (covering the period between December 2020 and March 2021).
Evaluation time point: At what time point are classifications into the three groups (positive tested cases, DPT, MCT) established? Shortly after the time of exposure, when PCR test results are still pending (and PCR+ denoting individuals who are later confirmed to be PCR-positive? Cross-sectional at a given moment in time?	Cumulative outcome events are analyzed. That is, any event is carried forward until the end of the follow-up observation period.
Case/outcome definitions: What is the accuracy with which infection status can be determined (i.e. how to deal with infected, untested individuals)?	All case definitions are based on self-reports by survey respondents.
- SARS-CoV-2 test	Having had an unspecified SARS-CoV-2 test in past 4 weeks in at least one follow-up.
- Positive SARS-CoV-2 test	Having had an unspecified positive SARS-CoV-2 test during past 4 weeks in at least one follow-up, but no such event before or at baseline.
- Exposure Notification	Having had an exposure notification in at least one follow-up, but no such event before or at baseline.
- Quarantine (or isolation)	Having been sent to quarantine by health authorities or a physician during past 4 weeks in at least one follow-up. Note: because the distinction between quarantine (of exposed contacts) and isolation (of infected individuals) are not widely known, the survey only asked for quarantine events (which respondents most likely understood to also include isolation of positive tested individuals).
Setting-specific assumptions: Country-specific simplifications may be warranted based on the Test-Trace-Isolate-Quarantine strategy.	In Switzerland, all PCR- or Antigen positive individuals are immediately reported to cantonal health authorities and placed in mandatory isolation. Therefore, segments A and B should remain empty.

Supplementary Table S3. Standardized questions on SwissCovid app use in the COVID-19 Social Monitor.

The SwissCovid App has been launched by the Swiss Federal Office of Public Health to warn smartphone users in case of possible exposure risks. The app records, if a contact has been in close proximity of 1.5m or less for longer than 15 minutes.<br>If an app user tested positive for the Coronavirus, she or he can anonymously notify other app users, who were in close proximity during the infectious period.	
	Are you using the SwissCovid App?<br>• Yes, permanently<br>• Yes, but sometimes I turn off Bluetooth to pause the SwissCovid App<br>• No, but I am planning to use it<br>• No<br>• No, I have uninstalled the SwissCovid App
Filter If **No or No, but..**:	Why are you currently not using the SwissCovid App?<br>• I have not heard about the app<br>• I don't think the app is useful for me<br>• I can't install the app (e.g., owing to technical difficulties or because I do not own an Android or iOS smartphone)<br>• I fear for my privacy and protection of my data<br>• Other reasons, comment field
Filter if **yes or yes, but**:	Were you ever notified by the SwissCovid App that you have been in close proximity to a Corona-positive person?<br>• No, I have never received a notification<br>• Yes, I called the recommended Infoline SwissCovid<br>• Yes, I sought testing for SARS-CoV-2<br>• Yes, I undertook other steps; comment field: which?<br>• Yes, but I did not undertake any steps
